# Evaluating deterministic motif significance measures in protein databases

**DOI:** 10.1186/1748-7188-2-16

**Published:** 2007-12-24

**Authors:** Pedro Gabriel Ferreira, Paulo J Azevedo

**Affiliations:** 1 Department of Informatics, University of Minho, Campus de Gualtar, 4710-057 Braga, Portugal

## Abstract

**Background:**

Assessing the outcome of motif mining algorithms is an essential task, as the number of reported motifs can be very large. Significance measures play a central role in automatically ranking those motifs, and therefore alleviating the analysis work. Spotting the most interesting and relevant motifs is then dependent on the choice of the right measures. The combined use of several measures may provide more robust results. However caution has to be taken in order to avoid spurious evaluations.

**Results:**

From the set of conducted experiments, it was verified that several of the selected significance measures show a very similar behavior in a wide range of situations therefore providing redundant information. Some measures have proved to be more appropriate to rank highly conserved motifs, while others are more appropriate for weakly conserved ones. Support appears as a very important feature to be considered for correct motif ranking. We observed that not all the measures are suitable for situations with poorly balanced class information, like for instance, when positive data is significantly less than negative data. Finally, a visualization scheme was proposed that, when several measures are applied, enables an easy identification of high scoring motifs.

**Conclusion:**

In this work we have surveyed and categorized 14 significance measures for pattern evaluation. Their ability to rank three types of deterministic motifs was evaluated. Measures were applied in different testing conditions, where relations were identified. This study provides some pertinent insights on the choice of the right set of significance measures for the evaluation of deterministic motifs extracted from protein databases.

## Introduction

The mining of *sequence patterns*, also called *motifs*, is one of the most important tasks in protein sequence analysis and continues to be an active topic of research. The large number of proposals found in the literature sustain this claim. Sequence mining is the task of analyzing a set of possible related sequences and detecting subtrings that occur significantly among those sequences. Motif over-representation can be explained by the existence of segments that have been preserved through the natural evolution of the proteins and suggests that the regions described by those substrings play a structural and functional role in the protein's mechanisms [1,2]. Different types of motifs representation have been proposed and two main classes can be distinguished: *probabilistic *and *deterministic*. A probabilistic motif consists of a model that simulates the sequences or part of the sequences under consideration. When an input sequence is provided, a probability of being matched by the motif is yielded. Position Weight Matrices (PWM) and Hidden Markov Models (HMMs) are examples of probabilistic motifs. Deterministic motifs are commonly expressed by an enhanced regular expression syntax, either matching or not the input sequences. This paper is devoted to the evaluation of significance measures for deterministic motif discovery in protein databases. A critical aspect of the motif analysis process is that due to the completeness nature of deterministic mining algorithms the number of extracted motifs is often very large. Not all these motifs are particularly interesting and most of them certainly arise by chance. Therefore, it is crucial to propose scoring methods to discriminate the relevant and significant motifs.

By itself, the definition of a significant motif is an interesting problem. One possible solution to assess this significance is to delegate this decision to a biologist. An expert would analyze the target proteins and decide which motifs have biological interest. As this approach is only feasible for small and medium scale experiments, an alternative is to automatically evaluate motifs according to their statistical or informative importance. As pointed by Hart et al. in [3], statistical significance is often correlated with biological significance and provides a meaningful criterion for the analysis of relevant motifs.

In addition to support a better understanding of the protein's structure and function, motifs have also a wide-range of other applications. They can be used to perform clustering [4], family classification [2,5-10], discovery of sub-families in large protein families [11], gene expression analysis [12,13] and the study and discovery of homology relations [5]. The selection of the appropriate measures for a specific problem depends on how well they adjust to the problem. In the literature, many measures of interest and significance have been proposed. How to choose the most appropriate significance measure is still an open question.

Similar to this problem is the discovery of significant association rules. In the work of Tan, Kumar and Srivastava [14], a survey and general evaluation of itemset interest measures is presented. Such measures were used to describe the statistical relationship between the items in a itemset [15]. This problem is different from the motif evaluation problem, since an item occurs only once per itemset, which is not the case of motifs, where an item (called symbol) may occur repeatedly. Transcription Factor Binding Sites (TFBS) can be described by motifs with very specific characteristics. Typically, they consist of small length contiguous motifs, highly degenerated, i.e., with many ambiguous positions. In Tompa *et al. *[16], an assessment of 13 popular algorithms for the discovery of TFBS was performed. Later, Li and Tompa [17] have categorized and examined the adequacy of three popular significance functions used by the algorithms described in [16].

Although, these studies were designed for problems other than protein motif analysis, they may bring important improvements to the field. For instance, the results of the unsupervised mining of massive protein datasets, such as the SwissProt [18] comprehensive protein sequence database, are almost impossible to be properly analyzed. This can be mainly due to the inexistence of measures that objectively and automatically evaluate the biological significance of newly discovered motifs and allow the identification of the truly significant motifs among the irrelevant ones.

Different measures evaluate different properties. Thus, the best solution for a particular problem may include the simultaneous use of several measures. Given that some of these measures will show consistent or even very similar results, it is important to identify such relations in order to avoid biased evaluations. We are also interested in studying the impact of different problem characteristics and how certain operations inherent to the mining process affect these measures.

The contributions of this paper can be summarized as follows:

• It surveys and categorizes significance measures presented in the bioinformatics, data mining, statistics and machine learning literature.

• It provides a comprehensive evaluation of the selected measures, in the presence of different motif and dataset characteristics.

• It proposes a methodology that combines the information provided by several measures in order to highlight the most interesting motifs.

The remainder of the paper is organized in two parts. In the first part we describe the characteristics of the evaluated motifs and the sources where the evaluated data is obtained. Significance measures are then introduced according to the considered categorization. The second part is dedicated to the experimental evaluation. We start by describing how motifs are extracted and then go on to the analysis of ranking, consistency and variability of the measures in a wide range of situations. In section "Motif Ranking Visualizer", we propose a methodology for identifying high scoring motifs and demonstrate its application. Finally, we conclude with the main lessons learned.

### Evaluating Deterministic Motifs

Deterministic motifs are described in a regular expression based language, which tends to be easily understandable by humans. These motifs can be divided in two types: *fixed-length *and *extensible-length*. Fixed-length motifs (a.k.a (*l, d*)-motifs [19,20]) consist of a string with a fixed size of *l *symbols where *d *possible symbols may have a mismatch with the matched sequences in the database. Extensible-length motifs have an arbitrary length with an arbitrary number of symbols and gaps. Consider the following abstract pattern:

*A*_1 _- *x*(*p*_1_, *q*_1_) - *A*_2 _- *x*(*p*_2_, *q*_2_) - ... - *A*_*n*_

*A*_*i *_is a sequence of consecutive amino acids, called *component *and -*x*(*p*_*i*_, *q*_*i*_)- represents a gap greater or equal than *p*_*i *_and smaller or equal than *q*_*i*_. A symbol is considered to be *concrete *if it represents one of the twenty amino acid symbols. Three types of extensible-length motifs can be distinguished:

• **Contiguous Motifs **contain no gaps, i.e., *p*_*i *_= *q*_*i *_= 0, ∀*i*, e.g. IPCCPV.

• **Rigid Gap Motifs **only contain gaps with a fixed length, i.e., *p*_*i *_= *q*_*i*_, ∀*i*. The symbol '.' is a wild-card symbol used to denote a gap of size one and it matches any symbol of the alphabet, e.g. *MN..A.CA*

• **Flexible Gap Motifs **allow a variable number of gaps between events of the sequence, i.e., *p*_*i *_≤ *q*_*i*_, ∀*i*, e.g. AN-x(1,3)-C-x(4,6)-D.

Deterministic motifs are typically mined through combinatorial algorithms that perform an exhaustive traversal of the search space and perform filtering using the support metric. The *support *of a motif is the number of different sequences where it occurs. For a motif to pass the filter, its support has to be equal or greater than a user pre-defined threshold (see [21-24] for a comprehensive overview). Support is an *apriori *measure of statistical significance. Generally, further assessment of motif significance is done as a post-processing step.

In this scenario, two important facts justify the critical need for the evaluation of significance measures. First it provides means for an early pruning of irrelevant motifs. The combinatorial nature of the deterministic mining process may deliver an exponentially increasing number of motifs. Thus, efficient pruning of irrelevant motifs results in performance improvement of the algorithms. Second, motifs over-representation does not necessarily imply significance.

In this work, three types of extensible-length motifs will be used to perform the evaluation of fourteen significance measures.

### The Prosite Database

There is a significant number of motif repositories freely available at the Internet. Examples of well established and reliable databases are: Prosite [25], Prints [26], Blocks [27], InterPro [28] or eMotif [29] (see [30] for an overview). From the listed databases, Prosite deserves a special attention in the context of our work. Prosite [25] is the oldest and best known sequence motif database. It is semi-manually annotated and its motifs are characterized for having a high biological significance. They provide a strong indication of a region in the protein with an important role. A family of protein sequences is then described by one or more motifs. Since this database is considered a standard, new algorithms and methods tend to use it as a benchmark test-bed.

### The Dilimot Database

One of the characteristics of the Prosite motifs is that they are strongly conserved in the respective families, covering the majority or the totality of their sequences. In order to perform an evaluation on less conserved motifs, we have used the Dilimot database [31]. It provides a service for finding over-represented, short (3 to 8 amino acids), rigid gap motifs in a set of protein sequences. Additionally, it makes available high-confidence pre-computed motif sets from different species. In this work, several motifs from human related proteins will be used.

### Significance Measures

As introduced by Brazma *et al. *[22], a significance measure can be defined as a function of the form: *f*(*M, C*) → ℝ, where *M *represents the motif being evaluated and *C *is a set of related proteins sequences usually called *target family *or positive data. This function returns a real value score that expresses how relevant or significant is *M *with respect to *C*. These scores may provide hints to biologically or statistically relevant motifs. If additional sequence information is available, for example where motifs are less expected to occur, both positive and negative information can then be considered in the evaluation. The function can be extended to include the negative dataset $\bar{C}$: *f*(*M, C*, $\bar{C}$) → ℝ. The universe of all sequences *U *corresponds to *U *= *C *+ $\bar{C}$ and the size of each set of sequences is denoted as |*C*| and |$\bar{C}$|, respectively. We now distinguish four possible cases of a motif *M *matching a sequence of *C*:

• *True Positive *(*T*_*P*_): a sequence that belongs to the target family and matches the motif.

• *True Negative *(*T*_*N*_): a sequence that does not belong to the target family and does not match the motif.

• *False Negative *(*F*_*N*_): a sequence that belongs to the target family and does not match the motif.

• *False Positive *(*F*_*P*_): a sequence that does not belong to the target family and matches the motif.

Sagot [32], suggests that motifs can be evaluated according to the following approaches: probability of matching a random sequence, sensitivity/specificity, information content and minimum description length (MDL). Since this categorization does not include all possible measures, nor distinguishes the type of information provided, a different categorization will be considered. Three categories are proposed:

1. *Class-based *measures, which are calculated based on the information of the motif in relation to positive and negative data.

2. *Information-Theoretic *measures, which are based solely on Information-theoretic models like probabilistic or entropy models. In this case the calculation is self-contained, i.e., the necessary information is found in the motif itself.

3. *Hybrid *measures use both Information-theoretic and class information.

### Class-based Measures

The ideal motif is one that matches all the sequences of the target family and no other sequence outside this family. It is also known as *signature *motif. In this context, the measures most widely used to express the quality of the motifs are: *sensitivity*, *specificity *and *positive predicted value *(see Table 1). Sensitivity (Sn), also called recall, measures the proportion of sequences of the target family correctly matched by the motif. Specificity (Sp) measures the proportion of sequences outside the target family that are not matched by the motif. Positive Predicted value (PPV), also called precision, measures the proportion of sequences that are covered by the motif and that belong to the target family. An ideal motif is one with 100% of Sn and PPV. These three measures yield a positive rank of motifs, i.e., their score is proportional to the rank. For comparison purposes, a negative rank measure *false positive rate *(Fpr) is also considered. This measure returns the proportion of negative instances that were incorrectly reported as being positive. In this case, the greater the score the worst the quality of the motif. Motifs can be ranked according to one or all of these measures. When a unique value is required to score a motif, a combination of these measures can be used. The *F-Measure *(F) [33] and the *Pearson Correlation *(Corr) [22,34] (also known as Matthews Correlation Coefficient, for its application in secondary structure prediction [35]) are examples of such composed measures. As a last example of a class-based measure we refer to the *Discrimination power *(Dp) [2]. This measure is particularly useful as a filter, since Dp is proportionally associated to selectiveness. A characteristic of class-based measures is that they do not rely on the motif structure to be calculated. Hence, they can be applied to any type of deterministic motif. Although a myriad of class-based measures can be found, covering different aspects of a pattern quality, we only review those widely used in a biological context. Please refer to Table 1 and 2 for details on these measures.

**Table 1:** List of the motif significance measures.

Symbol	Measure	Formula	Range	Type
Sn	Sensitivity	$Sn(M)=\frac{T_{P}}{T_{P}+F_{N}}$	[0,1]	C
Sp	Specificity	$Sp(M)=\frac{T_{N}}{T_{N}+F_{P}}$	[0,1]	C
PPV	Positive Predicted Value	$PPV(M)=\frac{T_{P}}{T_{P}+F_{P}}$	[0,1]	C
Fpr	False Positive Rate	$Fpr(M)=\frac{F_{P}}{F_{P}+T_{N}}$	[-1,1]	C
F	F-Measure	$F(M)=\frac{2\times Sensitivity\times PPV}{Sensitivity+PPV}=\frac{2\times T_{P}}{2\times T_{P}+F_{N}+F_{P}}$	[0,1]	1
Corr	Correlation	$C(M)=\frac{T_{P}\times T_{N}-F_{P}\times F_{N}}{\sqrt{\left(T_{P}+F_{N})(T_{P}+F_{P})(T_{N}+F_{P})(T_{N}+F_{N}\right)}}$	[-1,1]	C
Dp	Discrimination Power	$Dp(M)=\frac{T_{P}}{\left|C\right|}-\frac{F_{P}}{\left|\bar{C}\right|}$	[-1,1]	C
IG	Information Gain	$\begin{array}{l} IG\left(M\right)=Info\left(M\right)\times \left[Support\left(M\right)-1\right]\\ \text{where }Info\left(M\right)=-log_{\left|\Sigma \right|}p\left(M\right) \end{array}$	[0, + ∞[	IT
Pratt	Pratt Measure	$\begin{array}{c} Pratt(M)=\sum_{i}^{n}I^{\prime }(A_{i})-c\cdot \sum_{k=1}^{n-1}\left(q_{k}-p_{k}\right)\\ \text{where }I^{\prime }(A_{i})=-\sum_{a_{i}\in A_{i}}(P(a_{i})\times log(P(a_{i})))+\sum_{a_{i}\in A_{i}}\left(\frac{P(a_{i})}{P(A_{i})}\times log(\frac{P(a_{i})}{P(A_{i})})\right)\\ \text{and }P(A_{i})=\sum_{a_{i}\in A_{i}}p(a_{i}) \end{array}$	]- ∞, + ∞[	IT
LogOdd	LogOdd	$\text{Logodd}(\text{M})=log(\frac{\frac{Support(M)}{NumSeqs}}{P(M)})$	]- ∞, + ∞[	IT
ZScore	Z-Score	$\begin{array}{c} Zscore(M)=\frac{Support(M)-E(M)}{N(M)}\\ \text{where }E(M)=N_{resid}\times P(M)\text{ and }N(M)=\sqrt{N_{resid}\times P(M)\times (1-P(M))} \end{array}$	]- ∞, + ∞[	IT
J	J-Measure	$\begin{array}{c} J(C;M)=P(M)\times j(C;M)\\ \text{where }j(C;M)=P(C|M)\times log_{2}\frac{P(C|M)}{P(C)}+(1-P(C|M))\times log_{2}\frac{\left(1-P(C|M)\right)}{\left(1-P(C)\right)} \end{array}$	[0, + ∞[	H
I	Mutual Information	$\begin{array}{c} I(Q;M)=H(Q)-H(Q|M)\text{ where }H(Q)=-\sum_{q\in \{C,\bar{C}\}}P(q)\times log_{2}P(q)\\ \text{and }H(Q|M)=-P(M)\times \sum_{q\in \{C,\bar{C}\}}P(q|M)\times log_{2}P(q|M) \end{array}$	[0, 1]	H
S	Surprise Measure	$S(M)=Info(M)\times P(C|M)=Info(M)\times \frac{Support(M\in C)}{Support(M)}$	[0, + ∞[	H

Description of the fourteen significance measures according to the respective type (C = Class based; IT = Information-Theoretic based; H = Hybrid). For each measure the abbreviation symbol used throughout the paper, the formula and the respective range.

**Table 2:** Auxiliary formulas.

Formula	Range
$P(C)=\frac{T_{P}+F_{N}}{T_{P}+F_{N}+F_{P}+T_{N}}$	[0,1]
$P(C|M)=\frac{T_{P}}{T_{P}+F_{P}}$	[0,1]
$\frac{P(C|M)}{P(C)}=\frac{T_{P}\times (T_{P}+F_{N}+F_{P}+T_{N})}{\left(T_{P}+F_{P})\times (T_{P}+F_{N}\right)}$	[0,1]
$\frac{1-P(C|M)}{1-P(C)}=\frac{F_{P}\times (T_{P}+F_{N}+F_{P}+T_{N})}{\left(T_{P}+F_{P})\times (T_{N}+F_{P}\right)}$	[0,1]
$Info\left(M\right)=-log_{\left|\Sigma \right|}P\left(M\right)$	[0, + ∞[

List of auxiliary formulas used for the calculation of measures from Table 1. The respective range is also provided.

### Information-Theoretic Measures

When analyzing the probabilistic aspects of genetic sequences, one of two models can be adopted: a Markov or a Bernoulli model. In Markov models, the probability distribution of a given symbol depends on the *n *previous symbols, where *n *determines the order of the Markov chain [8,36].

In Bernoulli models, sequences are generated according to an independent identically distributed (i.i.d.) process. Therefore, the occurrence of a motif *M *in a given sequence is assumed to be an i.i.d. process [37]. This means that both the input sequences and the occurrence of the amino acids are independent. Protein sequences where motifs are sought to be found are often biologically related.

Although the independence of the positions along a sequence and in the motifs is not always verified, it can be considered reasonable to work under the assumption of an i.i.d. model [38]. The probability *P *of a motif *M*, in the form *A*_1 _- *x*(*p*_1_, *q*_1_) - *A*_2 _- *x*(*p*_2_, *q*_2_) - ... - *A*_*n*_, can be calculated according to formula 1.

*P*(*M*) = *P*(*A*_1_) × *P*(-*x*(*p*_1_, *q*_1_)-) × *P*(*A*_2_) × *P*(-*x*(*p*_2_, *q*_2_)-) × ... × *P*(*A*_*n*_)

Since the probability of matching any symbol from the alphabet (denoted by character '.') is one (*P*('.') = 1), then *P*(-*x*(*p*, *q*)-) = 1 and $P(A_{i})=\prod_{a_{j}\in A_{i}}P(a_{j})$. We consider that the probability of an amino acid *a*_*j*_, *P*(*a*_*j*_), is given by its frequency in the Swiss-Prot database [18]. If ambiguous positions occur in substring *A*_*i*_, then its probability is given by formula 2.

$$P(A_{i})=\prod_{a_{j}\in A_{i}}(\sum_{k=1}^{\left|A_{i}\right|}P(a_{j}k))$$

where *a*_*jk *_stands for the *k-th *amino acid in position *j *of the substring *A*_*i*_. For instance, the probability of the substring *A *- [*GC*] - · · - *V *is given by 0.0783 × (0.0693 + 0.0152) × 1 × 1 × 0.0671 = 4.44 × 10^-4^. *Support*(*M*) is the number of times that a motif M occurs in different sequences of the database. *Support*(*M *∈ *C*) corresponds to the number of sequences in family C where M occurs.

Information-Theoretic measures quantify the degree of information encoded in a motif. We provide examples of five of these measures.

*Information Gain *(IG) [39,40] is used to measure the amount of accumulated information by a motif in relation to an amino acid sequence. In this measure (see Table 1), the self-information content *Info(M) *(see Table 2) quantifies the information content associated with the motif, i.e., how likely is *M *to occur. (*Support*(*M*) - 1) gives the occurrence of motif M in the positive dataset. The minus one value of this component allows to easily reject motifs that trivially occur once.

The Minimum Description Length (MDL) principle applied in [11,38], is also an Information-theoretic measure and can be made equivalent to the IG measure. MDL is used to score the motifs and to measure the fitness of these motifs with respect to the input sequences. Assuming the hypothetical transmission of sequences, the idea is to measure how much can be saved in this transmission, if one knows about the presence of the motif. Neville-Manning *et al. *[38] demonstrated that *K *× *log*_2 _*P*(*M*) is the saving obtained from a motif *M *over *K *covered sequences, which is equivalent to the IG formula.

The *LogOdd *(LogOdd) measure provides the degree of surprise of a pattern. It compares the actual probability of occurrence (relative support value) with the expected probability of occurrence according to the background distribution. The formula presented in Table 1 is a variant of the LogOdd formula introduced in [36], which was first proposed to measure the significance of probabilistic patterns. This measure is particularly useful when comparing motifs with different lengths [17,41]. Both IG and LogOdd measures can be applied to all types of deterministic patterns.

The *Pratt *(Pratt) measure was introduced by Jonassen *et al. *[42] to rank extensible gap motifs obtained from the Pratt algorithm. Its value is calculated in two steps. In the first step, the information encoded by the motif is calculated. The second step corresponds to a penalty that is considered when gaps occur. The last measure used was the Z-Score measure. Although it is essentially a statistical measure, it was included in this group as it can be calculated based on the support, the motif information and the number of amino acids in the database (constant value). This measure can be used to filter out irrelevant motifs by selecting only those whose actual number of occurrences considerably exceeds its expected number. This criteria is based on the following biological motivation: if a motif occurs more than it is expected to occur by chance, then it should have a biological interest [3,37]. Z-Score is one of the most widely used measures for motif evaluation, see for example [37,43].

In the Z-Score formula (see Table 1), *Support*(*M*) denotes the actual number of occurrences, *E*(*M*) the expected number of occurrences of *M*, and *N*(*M*) the square root of the expected variance.

It was generally verified that statistically relevant motifs, discriminated through the Z-Score function, match functionally important regions of the proteins [37,43]. Another important conclusion obtained from [37] is that for over-represented motifs, the non-maximal motifs (which are contained in other motifs) have a lower degree of surprise than the maximal ones. This result is a good example that significance measures can be used as a clever mechanism to prune motifs not only after, but also before, their significance is computed. The minimum support criterion provides a way to detect those motifs that occur frequently. Significance measures, like Z-Score or IG, allow to detect motifs that although not frequent occur more than expected or that represent a high degree of information. Both criteria are complementary in the task of automatically retrieving significant motifs from a database. Please refer to Table 1 and 2 for details on these measures.

### Hybrid Measures

Considering measures that use both Information-theoretic and class-based features to determine the significance of a pattern, we selected two measures that are popular in the machine learning and data mining communities: the *J-Measure *(J) [44] and the *Mutual Information *(I), which is derived from the Shannon's entropy theory [34,45,46].

For a class space *Q *= {*C*, $\bar{C}$}, the component *H*(*Q*) of the *I *measure (see Table 1) provides the degree of information encoded by *Q*. Given a motif *M*, component *H*(*Q*|*M*) measures the amount of uncertainty remaining about *Q *after *M *is known. The difference *H*(*Q*) - *H*(*Q*|*M*) provides the expected information gain about *Q *upon knowing *M*.

The J measure is the product of two factors. The first factor, *P*(*M*), provides the prior probability of motif occurrence. The second factor, *j*(*C*; *M*), considers a target class *C *and its complement $\bar{C}$ and measures the goodness-of-fit of *M *with relation to class *C*. It is also called *cross-entropy *[47].

In addition, we redefine the *IG *measure to account for the distribution of motifs among the protein families, leading to the definition of a measure called *Surprise-Measure *(*S*). The *S *measure combines the information content (*Info*) of the motif *M *with the conditional probability of *M *matching a sequence (*s*) from the target class *C*. This probability is given by the relative occurrence of *M *in *C*, $\frac{Support(M\in C)}{Support(M)}$, which corresponds to the positive predicted value of *M*. It expresses the amount of information provided by the motif and its quality as a class descriptor.

These three measures can be easily calculated for all types of deterministic motifs. In general, one can interpret such measures as a way to quantify the uncertainty reduction of a sequence *s *belonging to the class *C*, given that *s *contains the motif *M*.

In conclusion, the presented measures can be calculated based on two components of motif information: the class match information (*T*_*P*_, *T*_*N*_, *F*_*P*_, *F*_*N*_) component and the motif probability and gap information component. Class-based measures are calculated according to the first component, Information-theoretic measures based on the second and hybrid measures based on both. Table 2 contains formulas to support a better understanding of Table 1.

### Evaluation

We start by describing the algorithms applied to mine the three different types of motifs used in the experiments.

To mine contiguous motifs we developed a simple algorithm based on the n-gram methodology. A *n-gram *is a word of *n *contiguous symbols. The algorithm takes as input a set of sequences and the *target motif*, which represents the motif to be primarily spotted. It extracts words with the length of the target motif (n = motif length) through window sliding. Each word is hashed into a table and the respective support count incremented. Finally, the score values for the different measures of all the scanned words are calculated. Due to their popularity within the bioinformatics community, Teiresias [48] and Pratt [49] were used to extract rigid and flexible gap motifs, respectively. Besides the input dataset, Teiresias algorithm accepts as input three parameters: minimum support, L and W, where L defines the minimum number of concrete symbols that a word of length W must contain. Pratt allows specifying the characteristics of the extracted motifs by setting a large number of parameters. It automatically scores the motifs according to the Pratt measure. With the exception of the minimum support value and the number of reported motifs all the remaining Pratt parameters were used assuming the default values recommended by the authors (program available at [50]). Additional details for the use of these programs are provided whenever necessary.

The consistency between two measures can be defined as follows:

**Definition 1. (Measure Consistency) ***Given two measures M*_1_* and M*_2_* and the respective score value vectors V*_*M*1_* and V*_*M*2_,* the respective consistency is determined by the Pearson's Correlation between its vectors, corr*(*V*_*M*1_, *V*_*M*2_).

Informally, a motif is considered to be strongly conserved if it occurs in the majority of the input sequences, i.e., its relative support value is approximately 100%. Alternatively, it is considered weakly conserved if its relative support is considerably below 50%.

### Ranking Analysis

In this first experiment, the ability of the introduced measures in ranking the three different types of motifs is evaluated. The general evaluation procedure was as follows: select a target motif from Prosite, Dilimot or synthetically generated motif. Gather the set of related protein sequences where false negatives may occur. The parameters of the algorithm are refined until the target motif is included in the reported solution. For motif ranking evaluation only positive information is considered. Since not all the elements of class match information are available, only Information-theoretic measures are used in the ranking evaluation. In order to assess the quality of the measures in ranking the target motifs, a metric called *R*_*m *_(Formula 3) was used, where *N*_*motifs *_is the total number of evaluated motifs and *Rank*_*motifs *_the sum of the respective rank values. Measures with *R*_*m *_closer to 1 are the best.

$$R_{m}=\frac{N_{motifs}}{Rank_{motifs}}$$

#### Contiguous Motifs

Real protein sequence data was obtained from Prosite. Entries that contain contiguous motifs were selected and the respective sets of sequences retrieved. Additionally, synthetic protein data was generated. Each synthetic dataset consists of 50 sequences of length 300. For each dataset, a motif of a given length was randomly generated and planted once in all its sequences. The generation of sequences and motifs was done according to the Swiss-Prot amino acid frequency. Motifs were then extracted according to the described n-gram methodology.

Table 3 shows the ranking of 11 Prosite motifs and Table 4 the results for a group of 8 synthetic protein datasets. In both cases, the target motifs are highly conserved with a support of around or equal to 100%.

**Table 3:** Evaluation of contiguous motifs on Prosite data.

PS entry	Motif	NumSeqs	DiffNGrams	Rel. Supp(%)	Supp Rank	ZScore	LogOdd	Pratt	IG	Info
PS00341	IPCCPV	9	702	77.8	9	21	65	166	13	217
PS00415	LRRRLSDS	12	3582	91.6	9	503	1058	2103	11	1784
PS00047	GAKRH	105	653	93.3	21	61	109	216	27	460
PS00984	CFWKYC	19	1256	100	1	1	1	785	1	5
PS00541	SKRKYRK	6	144	100	1	85	110	131	3	134
PS00822	PFDRHDW	9	2251	100	1	1	5	204	1	400
PS00419	CDGPGRGGTC	207	32936	100	1	1	1	3	1	158
PS00349	RKRKYFKKHEKR	18	2929	100	1	38	86	2884	19	310
PS00861	GWTLNSAGYLLGP	32	888	100	1	66	301	179	1	569
PS01024	EFDYLKSLEIEEKIN	60	5527	100	1	620	2427	5266	1	5244
PS00291	AGAAAAGAVVGGLGGY	136	2423	100	1	1033	1770	184	3	1984

*R*_*m*_					0.2340	4.526E-3	1.854E-3	9.075E-4	0.1358	9.764E-4

Ranking results of eleven Prosite datasets (identified by the Prosite (PS) entry column). For each dataset, the number of protein sequences, the number of different n-grams (Diff NGrams), where *n *is equal to the motif length and the relative support of the target motifs (Rel. Supp) are presented. Motifs are ranked with Information-theoretic based measures. Ranks obtained by support (Supp Rank) and information gain (Info) are also provided for comparison purposes. Last row gives the *R*_*m *_values of each measure, where best results are obtained by support and IG.

**Table 4:** Evaluation of contiguous motifs on protein synthetic data.

Motif	Supp	ZScore	LogOdd	Pratt	IG
SSN	1	3710	1	2130	1
IYKQ	1	1533	2	11817	1
NDFNE	1	1	1	13483	1
PLMPES	1	1	2	4973	1
MRKMVTAG	1	1	6	9818	1
TKYEETGAFK	1	1	43	7350	1
DRTGMHSIFFLP	1	1	3	11721	1
MTENKVGESICPAAPN	1	1	29	9589	1

*R*_*m*_	1	0.0015	0.0919	1.128E-4	1

Ranking results for eight synthetic protein datasets. Each dataset contains 50 sequences of length 300. Target motifs have a support of 100%. Motifs are ranked with Information-theoretic measures and support. Last row gives the *R*_*m *_values of each measure, where the best results are obtained by IG and support.

#### Rigid Gap Motifs

Table 5 shows the ranking of rigid gap motifs from ten datasets of the Dilimot database. This experiment was performed to evaluate weakly conserved motifs. Table 6 presents the results for 8 datasets from Prosite. The evaluation is focused on long and strongly conserved rigid gap motifs. Teiresias algorithm was used to extract the motifs, were L and W parameters were set to conform the characteristics of the target motif and the minimum support set to 80% of its actual support.

**Table 5:** Evaluation of rigid gap motifs on Dilimot datasets.

Motif	NumSeqs	Abs. Supp	Supp Rank	IG	Pratt	LogOdd	Zscore
LPSN	15	4	1294	520	2429	4	6
WS.WS	34	7	15	22	31	28	28
Q.RLQ..Q	15	4	5259	660	5213	1	1
P.LP.K	24	8	1334	336	592	22	23
L.DL.K	7	7	1	1	12	1	1
M.C..S.E.K.A	5	4	101	14	424	17	17
GS...G.P	25	5	22554	10428	11292	1155	1243
G...E.GE	40	9	4735	1257	3617	30	32
R.RS.S	32	6	3497	1319	1395	42	52
G...RGRG	15	8	97	1	136	1	1

*R*_*m*_			0.0003	0.0007	0.0004	0.0077	0.0071

Evaluation of motif ranking results for ten datasets from the Dilimot database. For each dataset the number of sequences and the absolute support value (Abs. Supp.) of the target motif are given. Motifs are ranked with Information-theoretic measures and support (Supp rank). Last row gives the *R*_*m *_values of each measure, where LoggOdd obtained the best results.

**Table 6:** Evaluation on rigid gap motifs on Prosite datasets.

PS entry	Motif	Total Motifs	NumSeqs	Abs. Supp	Supp Rank	IG	Pratt	LogOdd	Zscore
PS00084	HHM..F.C	206	13	10	1	4	54	3	3
PS00927	PGGRF.E.Y.WD.Y	60	32	32	5	2	1	2	2
PS01142	GTLW.G...........L....W	419	5	4	1	3	198	3	3
PS00780	NHT.C.C.TC..HK	30	57	54	8	7	3	9	9
PS00799	C.D..HCCP....C	285	6	5	1	53	91	50	50
PS00987	GKCNN..GHGHNY	106	13	6	1	4	94	3	3
PS00458	P...LGP.C.Y.AA.V.R...HW..P.L.AGA.A.G...K	579	11	11	1	1	1	1	1
PS00506	H.CGGNVGD	41	16	15	14	2	27	2	2

	*R*_*m*_				0.25	0.11	0.0171	0.1096	0.1096

Evaluation of motif ranking results for eight datasets from the Prosite database. For each dataset the number of sequences, the absolute support value (Abs. Supp.) and the number of reported motifs are given. Motifs are ranked with Information-theoretic measures and support (Supp rank). Last row gives the *R*_*m *_values of each measure, where support obtained the best results.

#### Flexible Gap Motifs

For flexible gap motifs, a slightly different experiment was performed. In this case, it was evaluated how Information-theoretic measures relate to the Pratt measure.

The Pratt algorithm was used to extract 250 flexible gap motifs from the Prosite dataset entry PS00034 (55 sequences). The characteristics of the reported motifs (consider the definition of Extensible-length motifs in section "Evaluating Deterministic Motifs") range from 50% to 100% for the support value, from 4 to 9 for the number of concrete symbols and from 1 to 8 to the number of components.

## Discussion

In the evaluation of contiguous motifs, n-grams of the length of the target motif were extracted. When all the evaluated motifs have the same length, measures that are mainly based on the information embedded by the motifs provide very poor results. This can be confirmed in Table 3 and 4 by the results of the Pratt measure, essentially based on information gain. In Table 3, we also present the ranking results provided by the self-information (Info) component as described in Table 2, which represents additional confirmation of this result. The main reason for such bad results is that Pratt provides roughly the same score for all the contiguous motifs, since they have the same length and only one component.

Introducing the support as a criterion to score the motifs improves the quality of the ranking results. Support provides an important motif discrimination feature. This is confirmed by the results of the support, IG and Z-Score measures.

**Figure 1:**
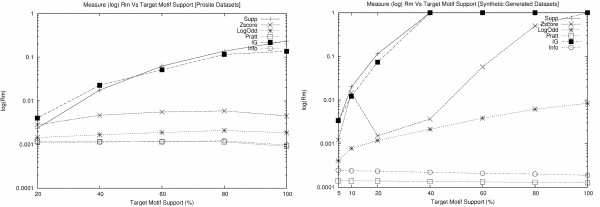
**Ranking performance for different support values of the prosite and synthetic datasets**. These figures presents the variation of the *R*_*m *_metric for each measure and according to different support values of the target motif. *R*_*m *_is presented in logarithmic scale (y-axis) and support in relative values (x-axis). Evaluation performed for the prosite and synthetic datasets from Table 3 and 4. Support, IG and Z-score have, respectively, the best results for the two sets.

Target motifs appear highly conserved in the datasets from Table 3 and 4 and consequently experiments can be biased in favor of support. An additional experiment was devised where the support of the target motifs was reduced for different values. This was done by removing from the dataset the appropriate number of motif occurrences. Rank results were then obtained, both for prosite and synthetic datasets, and presented in Figure 1. It can be seen from these two experiments that even for lower support values Support and IG still maintain a clear advantage over the remaining measures.

The main conclusion that can be drawn from this first evaluation is that when motifs have very similar characteristics regarding their length and composition, support or measures mainly based on support are the most appropriate for motif ranking.

Table 5 presents the results for the ranking of weakly conserved motifs. Here, Z-Score and LogOdd have a very similar behavior, producing the best results. Support-based measures are not suitable in this situation as many motifs have a higher support than the target motif and therefore will have a better rank.

For situations where a low minimum support threshold is used (below 50%) and where the reported motifs occur within a wide range of support values, measures that provide their score based on the deviation between the actual and the expected number of occurrences seem to be the most appropriate.

For strongly conserved rigid gap motifs, presented in Table 6, and as already verified with contiguous motifs, support and support-based measures as the IG, LogOdd and Z-Score are good enough to discriminate the target motifs. It is interesting to note that these last three measures provide very similar results and that Pratt also has reasonable results. Note that for Prosite entry PS00799, the three measures IG, LogOdd and Z-Score provide a bad result. A closer analysis to this dataset has shown that the target motif is contained in another nine longer motifs and that the first five of these motifs were ranked at positions 1, 3, 10, 15, 28.

The impact of motifs features, namely support, length (number of concrete symbols), number of don't care symbols and number of components on each of the Information-theoretic measures was also evaluated. We have collected the 1726 motifs for all the datasets described in Table 6. The following observations can be made by quantifying the consistency between features and measures, and between measures:

• The feature "number of don't cares" does not seem to have a significant impact in any of the measures since all the respective correlations are smaller than 0.3.

• LogOdd and the logarithm of Z-Score show a clear linear relation.

• The length has the biggest impact in the LogOdd and consequently in the log(Z-Score). The consistency with these two measures is approximately 0.5 and for the other measures less than 0.4.

• The consistency of support is very high with IG (~0.8) and very low with the remaining measures.

• The feature "number of components" has a very high consistency with Pratt (~0.85) but also high with LogOdd (~0.62) and Z-Score (~0.4). The first relation can be explained by the fact that the Pratt measure was designed to score motifs with several components and for each component a fixed value is given. Thus, the greater the number of components the higher the Pratt scores. The second case is a consequence of the fact that the number of components is proportional to the length of the motifs and as already observed LogOdd and Z-Score are consistent with this feature.

**Figure 2:**
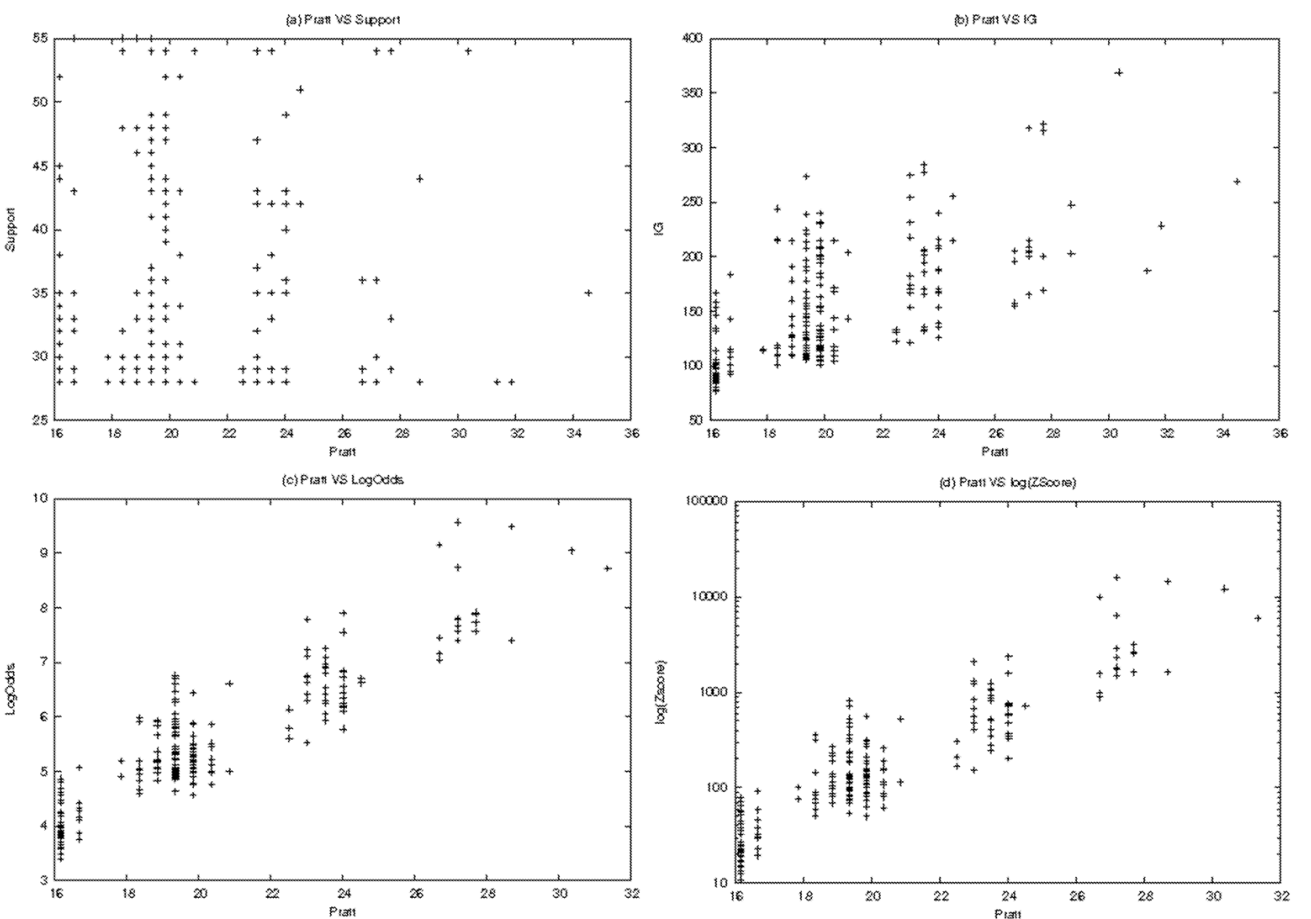
**Plot Between Pratt measure and four measures**. The Pratt algorithm was used to extract 250 flexible gap motifs from the Prosite dataset entry PS00034 (55 sequences). The characteristics of the reported motifs range from 50% to 100% for the support value, from 4 to 9 for the number of concrete symbols and from 1 to 8 for the number of components. The plots from this figure depict the following relations between: (a) Pratt and Support; (b) Pratt and IG; (c) Pratt and logOdd; (d) Pratt and logarithm of Z-Score.

The four plots from Figure 2 depict the relation of Pratt with support, IG, LogOdd, Z-Score in the evaluation of 250 flexible gap motifs from the Prosite family PS00034. The plot in Figure 2 (a) shows clearly that Pratt has no relation with support. The Pratt measure does not take into account the number of sequences matched by the motif when evaluating its significance. The authors [42] assume that since all the reported motifs respect the minimum support value, they are in the same conditions and therefore only the information provided by the motif composition is considered. This explains many of the poor results of Pratt in motif ranking (from Table 3 to Table 6). For this dataset, Pratt and IG have a relatively high consistency (approximately 0.6; see Figure 2 (b)). Both measures make a strong use of the information gain provided by the motif composition. Pratt is highly consistent with LogOdd and logarithm of Z-Score (see Figure 2 (c, d)). This results from the fact that these three measures are proportional to the length of the motifs.

Pratt was designed to score motifs with several components, a substantially different structure among them and small support variations. In cases where motifs have only one component (contiguous motifs) or roughly the same structure, for example A.A.A.S and P.P.P.S, it scores roughly in the same way all the motifs, which makes difficult to distinguish the truly significant ones. The same also happens when motifs have considerably different support values.

An important conclusion from our evaluation is that it is very important that a score measure always take into account the support (relative or absolute) of the motifs. This provides an essential criterion to distinguish the significant motifs from the background model and among each other.

### Consistency Analysis

Consistency provides a way to express the degree of redundancy among the information provided by the measures. In the previous section we have already presented some results relative to this topic for Information-theoretic measures. In this section, we extend this study to all the measures. Positive and negative sequence information is considered and experiments testing different conditions are performed. The first experiment is intended to describe a generic situation where motifs have no specific characteristic and therefore their properties vary in a wide range of values. The second experiment evaluates how measures react to three possible motif operations.

To execute these experiments a generator of motif meta-information was developed. For each motif, meta-information consisting of tuples (*probability*, *numGaps*, *T*_*P*_, *T*_*N*_, *F*_*P*_, *F*_*N*_) is generated. These values are randomly generated according to the given range limits for each experiment and described in the following sections. The simulated dataset of positive information consists of 50 sequences and the negative dataset of 100 sequences, both with length 300. The choice of the datasets size was made to guarantee a conservative evaluation, by providing negative information a greater weight.

#### Generic Situation

**Figure 3:**
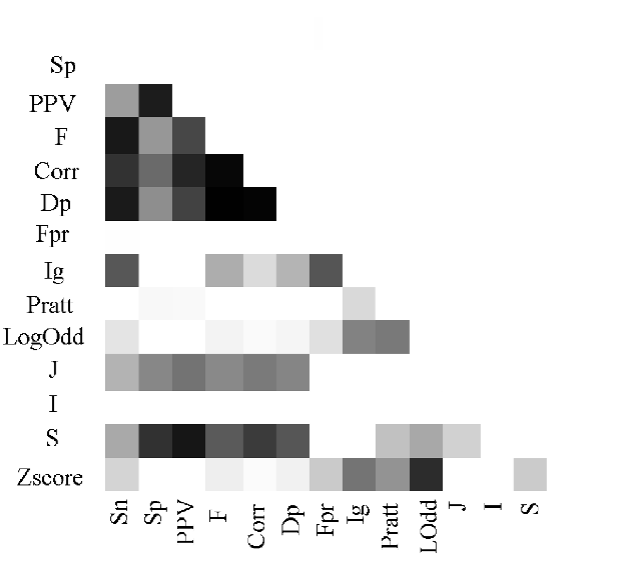
**Correlation Matrix for the 1000 motifs**. Correlation matrix of the 14 measures in the evaluation of the 1000 motifs. Parameters for the synthetic generation of the motifs: *T*_*P *_∈ [15, 50] (50 sequences for the positive dataset); *F*_*P *_∈ [0, 35] (100 sequences in the negative dataset); Length ∈ [5, 7] amino acid symbols and numGap ∈ [0, 5]. Dark areas indicate a higher correlation between the respective measures. Due to the symmetric nature of the matrix only the lower triangular part is presented.

**Figure 4:**
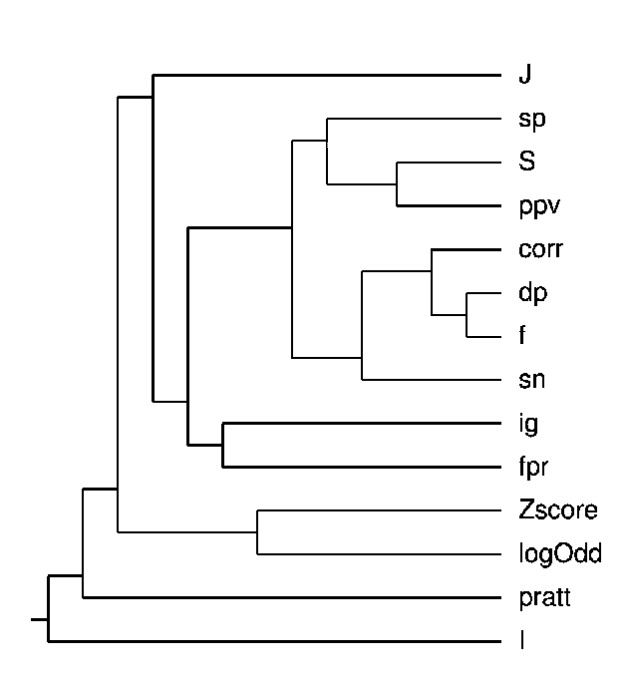
**Dendogram for the 14 measures with the 1000 motifs**. The dendrogram depicts in a tree format the consistency between the fourteen measures. Closer distance in the tree represents higher consistency.

For this experiment 1000 motifs were generated with the following parameters: *T*_*P *_∈ [15,50]; *T*_*N *_∈ (50 - *T*_*P*_) (50 sequences for the positive dataset); *F*_*P *_∈ [0, 35]; *F*_*N *_∈ (100 - *F*_*P*_) (100 sequences in the negative dataset); Length ∈ [5,7] amino acid symbols and numGaps ∈ [0, 5]. Figure 3 shows the correlation matrix for the 14 measures. Each measure is associated to a vector of values (1000) and an all-against-all vector comparison is made with the respective correlation being calculated. Dark areas indicate a high correlation, and according to Definition 1 a higher consistency. Figures from the Additional File 1 shows the correlation matrices according to different values of sensitivity for the 1000 motifs. Figure 4 presents a dendrogram that depicts measure consistency for the 1000 motifs case.

From these results the following observations can be made: Sn is highly consistent with F, Corr, Dp, in particular for smaller values, i.e., Sn ≤ 33% (please note the first column of the matrices from Figure 3 and Additional File 1). This confirms that these three measures are also good indicators of motif over-representation. F, Corr, and Dp are also highly consistent with Sp and PPV, which demonstrates that they are equally good replacing Sn, Sp and PPV when a unique score value is required.

Fpr and IG show high correlation mainly due to the fact that both measures have a small variation. The S and J measures show a strong correlation with Sp, PPV, F, Corr and Dp for smaller values of Sn. The correlation becomes weaker for higher values of Sn. The consistency of the S measure with class-based measures is naturally expected since S includes in its formula the PPV value, that is highly correlated with all the class-based measures. Regarding the J measure, we have evaluated independently the effect of its two components, *P*(*M*) and *j*(*C*; *M*). It was verified that the consistency of these components with Sp, PPV, F, Corr and Dp is smaller than with the original measure. Thus, we can conclude that the results for the J measure are not biased by any of its components and that the overall effect of the measure is able to determine the quality of the motifs as class descriptors. The relation between LogOdd and Z-Score is again confirmed by the strong consistency between these two measures.

#### Motif Operations

Essentially, deterministic motif mining algorithms make use of three operations: *generalizations *(a concrete symbol is replaced by don't care or set of symbols), *specializations *(don't care symbols are replaced by concrete symbols) and *extensions *(concrete symbols are appended to the motif). In this experiment, we study the impact of these three types of operations in the studied measures. For each operation meta-information for 100 motifs was generated according to the variables and values described in Table 7. The positive dataset consists of 50 sequences and the negative dataset of 100 sequences. To simulate generalization operations, which typically corresponds to an increase in the support, motifs are generated for a range of high *T*_*P *_values. Specializations are simulated equivalently, but for low values of *T*_*P*_. Different motif lengths are generated to simulate the extension operation.

**Table 7:** Parameters values for the simulation of three motif operations.

Operation	*T*_*P*_	*F*_*P*_	Length	Num gaps
Generalization	[25, 50]	5	5	[1, 4]
Specialization	[5, 25]	5	5	[1, 4]
Extension	40	5	[5, 10]	[1, 9]

Three motif operations – generalization, specialization and length extension – are simulated through the generation of motif information with different parameter values. Generalization and specialization are controlled through the true positive rate, with a fixed false positive and fixed number of concrete symbols (length). Extension is controlled by the length parameter. The number of gaps is calculated for the respective range and proportionally to the length of the motif.

Table 8 presents the correlation between Sn (that represents generalizations and specializations), motif length extension and the remaining measures. Besides class-based measures, the IG measure has the best linear relation with the three operations. This results from the fact that IG is composed of two components: absolute support, which is directly proportional to Sn and information content (Info) provided by motif composition that is proportional to the length (number of symbols). The S measure also has a considerable consistency due to the same reasons pointed for the IG measure.

**Table 8:** Correlation of the measures with the three simulated operations.

Operation	Sp	PPV	F	Corr	Dp	Fpr	IG	Pratt	LogOdd	ZScore	J	I	S
Generalization(Sn)	-	0.99	0.99	1.0	1.0	-	0.92	0.10	0.05	0.22	0.18	0.1	0.24
Specialization(Sn)	-	0.96	0.99	0.99	1.0	-	0.97	0.11	0.26	0.26	0.39	0.2	0.82
Extension(Length)	-	-	-	-	-	-	0.99	0.69	0.99	0.71	-0.99	0.1	0.99

Impact of the three motif operations (described in Table 7) measured by the correlation of the significance measures with the values of Sn and length. The former is used to simulate generalizations and specializations and the latter extensions to the number of concrete symbols of the motif. For measures with constant score values no correlation value is provided.

LogOdd, Z-Score and Pratt are essentially affected by the length of the motifs, which is a confirmation of the results already discussed in section "Motif Ranking". The strong consistency of these three measures, as well as IG and S, with motif length, provides evidence that they can successfully discriminate motifs of different lengths. With the exception of one case (Prosite motifs; see Figure (d) from Additional File 1), the I measure has no consistency with other measures. This proves that, in general, this measure is not suitable for motif evaluation.

#### The Prosite case

The motifs in Prosite database have been used to evaluate measure consistency over real data. For this purpose only flexible-length motifs were evaluated. The file *Prosite.dat *that corresponds to the Prosite database (available by FTP) was analyzed, corresponding to the release 19.20 (Feb-2006). This release contains 1929 entries, where 1330 are regular expression motifs and 1317 entries contain class based information. The number of rigid gap motifs is 1030. The average PPV is 95.92% and the average Sn is 90.16%. The overall average gap length of the motifs is 1.93 with a standard deviation of 1.52. The Swiss-Prot database [18] (release 49.0) was used as the negative information. This database contains more than 8 millions amino acids for a total of 207132 non-redundant protein sequences.

Figure (d) from Additional File 1 presents the correlation matrix for this experiment, which corresponds to the evaluation of high quality motifs (high Sn and PPV), with variable length. Nevertheless, besides the high correlation between IG and I, and LogOdd and S, no significant differences with the previous experiences are detected. This seems to indicate that measures show a steady behavior for a wide range of cases.

For highly imbalanced situations, as the one exemplified by the Prosite experiment, where the negative dataset is significantly larger than the positive dataset, measures that make use of negative information, like Fpr and Sp, are of little use. The analysis of the Fpr scores shows that all motifs score closer to zero. This negative rank is not suitable for such cases, since no discrimination among the motifs can be obtained. In the same way, Sp will always show high scores due to large *T*_*N *_values.

#### Principal Component Analysis

The Principal Component Analysis (PCA) [51,52] technique was used to summarize and discover patterns of inter-correlations among the studied measures. This method describes the variation of a set of correlated variables in terms of a set of uncorrelated combinations, called principal components. These components, which express combinations of the original variables, allow a dimensionality reduction while maintaining as much as possible the variability of the original data.

This method was applied to the Prosite dataset described in the previous section. Fourteen components were obtained, where 4 have an initial eigenvalue greater than 1. The first four components show the highest percentage of variance and account for a cumulative variance of 89.1%. We have applied a rotation to the component matrix, according to varimax method with Kaiser Normalization [52]. Using a threshold value of 0.5, the following components were obtained: *C*_1 _= {*LogOdd*, *S*, *Z-Score*};

*C*_2 _= {*Sn*, *F*, *Corr*, *Dp*}; *C*_3 _= {*Sp*, *PPV*, *Corr*} and *C*_4 _= {*IG*, *I*}.

*C*_1 _relates measure LogOdd and Z-Score, where a clear relationship can be found since both provide a degree of emergence of the pattern, i.e., how much its support deviates from what was expected. These two measures are also correlated with the S measure, which combines information content (Info) with PPV, that also expresses motif over-representation.

*C*_2 _and *C*_3 _relates only class-based measures, where F and Corr measures are present in both components. This is due to the high inter-correlation between class-based measures.

*C*_4 _is more surprisingly interesting. It relates IG and I which are apparently two completely different measures. Although both measures combine information gain with class-based information, this is done in different ways. The combination of the fact that evaluated motifs are strongly conserved and the highly class imbalance of data may explain the biased results of these two measures.

### Variability Analysis

**Figure 5:**
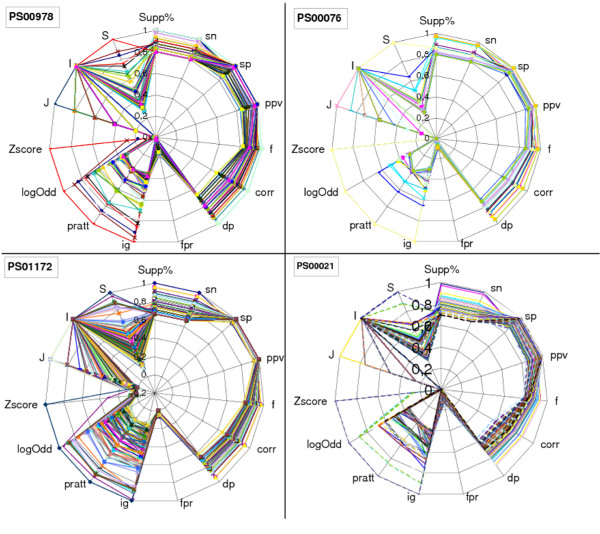
**Variability analysis for 4 Prosite families**. Variability analysis of the fourteen significance measures for four Prosite family entries: PS00978, PS001172, PS00076 and PS00021. For each family dataset, rigid gap motifs were extracted with a minimum support value equal to 80% of the Sn value of the Prosite target motif (signature motif of the family). For easier visualization, score values were normalized to the [0, 1] range. The number of evaluated motifs was respectively for each family: 94, 196, 20 and 88. Each line in the figure (plotted with different colors) represents the scores of a motif. Measures where the majority of their lines overlap have low variability. It can be seen that in general non class-based measures have greater variability.

The mining process typically reports a large number of motifs. Therefore, an important property of significance measures is its relative variability. Measures that provide a larger variability will allow an easier discrimination between high scoring motifs. We have studied several protein families from Prosite. For each Prosite family, rigid gap motifs were extracted, evaluated according the fourteen measures and normalized for easier visualization. Figure 5 depicts the variability of the measures of four Prosite family entries. Table 9 shows a different view of the variation analysis for all the 1330 Prosite motifs. The average, standard deviation and the coefficient of variation [51] are shown. From this table it can be observed that Z-Score shows an extremely large variation, due to the presence of very long motifs, with a very small probability of occurrence. Thus, even for a slight deviation between the actual and the expected support, to long motifs typically corresponds large Z-Score values.

**Table 9:** Variability values for the fourteen measure on the Prosite dataset.

Measure	Avg	Std	$\frac{Std}{Avg}$
Sn	0.910	0.122	0.134
Sp	1.000	0.000	0.000
PPV	0.968	0.091	0.094
F	0.931	0.099	0.106
Corr	0.935	0.091	0.097
Dp	0.919	0.122	0.132
Fpr	0.000	0.000	0.000
IG	552.031	755.787	1.373
Pratt	20.763	13.088	0.631
LogOdd	3.736	3.002	0.817
J	-8.888	3.119	0.359
I	0.005	0.007	1.400
S	7.467	2.612	0.349
ZScore	3 M	124 M	41.3 M

Average, standard deviation and coefficient of variation of the 1330 rigid gap Prosite motifs.

In general, one can say that class-based measures show small variability, which in this case is a consequence of the high quality of Prosite motifs (high Sn and PPV).

### Motif Ranking Visualizer

Evaluation in section "Ranking Analysis" shows that significant disagreements between ranking results of the different measures occur frequently. If the choice of the right scoring criterion is not clear, the use of several significance measures can be an alternative. This option leads to high confidence results when the different measures are in accordance, but may lead to difficulties in identifying the most interesting motifs when disagreements are verified. The example depicted in Table 10 illustrates this situation. Motif A scores higher using measure 1, B using measure 2 and G with measure 3. Apparently, these seem to be the most interesting motifs. A closer look at Table 10 shows a very small variation for measure 3. Although, G scores higher, the remaining motifs have similar scores. Also, motif D has good performance on the three measures, representing an example of a motif that should also be spotted.

**Table 10:** Example of motif scoring for three measures and respective values range.

Motif	Meas.1	Meas. 2	Meas. 3
A	0.92	0.39	0.78
B	0.1	1.0	0.83
C	0.05	0.35	0.82
D	0.8	0.8	0.83
E	0.2	0.2	0.82
F	0.4	0.3	0.84
G	0.1	0.2	0.85
H	0.15	0.14	0.81

Range	[0.05; 0.92]	[0.14; 1.0]	[0.78; 0.85]

This table exemplifies the hypothetic scoring of eight motifs according to three significance measures, where not only the high scoring motifs of each measure are the most interesting. Last row provides the range of the score for each measure.

If several measures are applied for motif scoring, three attributes that contribute to a better motif filtering can be considered. We now describe each attribute in detail, how they can be calculated and demonstrate with an example the application of the proposed methodology. The three attribute values are scaled to fit the [0,1] range. Consider a motif M and the respective score vector [*D*_1_(*M*), *D*_2_(*M*), ⋯, *D*_*n*_(*M*)] for the *n *scoring measures. The first attribute describes the frequency of the motif in the positive dataset, i.e., its support. This characteristic is important since it provides an *apriori *criterion of motif significance and is easily obtained by any motif mining algorithm. The second attribute indicates the average motif ranking position for the *n *applied measures. This is provided by the *maxValue *function, described by Formula 4.

$$maxValue(M;D_{1}(M),D_{2}(M),\cdots ,D_{n}(M))=\frac{\frac{D_{1}(M)}{max(D_{1})}+\frac{D_{2}(M)}{max(D_{2})}+\cdots \frac{D_{n}(M)}{max(D_{n})}}{n}$$

A motif with the highest score in all the measures has a maxValue of 1. As verified in the example of Table 10 (see measure 3), a motif may score higher in a certain measure. However, if this measure has a small variability, the amount of information gain obtained with such score is low. The third attribute describes the amount of surprise/information gain that results from the motif score when compared with the remaining scores for the respective measure. This can be estimated through the average of the normalized scores for all the measures. The information gain score is given by Formula 5.

*info*(*M*; *D*_1_(*M*), *D*_2_(*M*), ⋯, *D*_*n*_(*M*)) = *N*(*D*_1_(*M*), *D*_1_) + *N*(*D*_2_(*M*), *D*_2_) + ⋯ *N*(*D*_*n*_(*M*), *D*_*n*_)

where *N *corresponds to min/max normalization of *x*, given by $\frac{x-min}{max-min}$. *min *and *max *are the minimum and maximum values for each vector *D*_*i*_. *D*_*i*_(*M*) is the score value of motif *M *for measure *i*.

**Figure 6:**
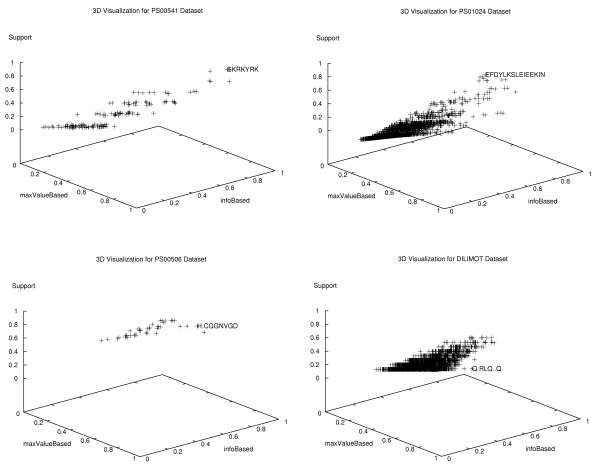
**Three dimensional visualization of motif scoring**. Three-dimensional visualization of motifs from the datasets: PS00541, PS01024, PS00056 and DILIMOT "Q.RLQ..Q". The first two datasets contain respectively 144 and 5527 contiguous motifs and the last two 41 and 5371 rigid gap motifs. Motifs are scored with the attributes: support, maxValue and infoBased. Target motifs can be easily spotted among: the ones that are highly ranked in two or three dimensions, the ones which significantly deviate from the majority of the other motifs, or the ones that appear in the border regions of motif clouds.

In order to test the ability of our methodology for spotting the most interesting motifs, we have applied it to some of the previously evaluated datasets. Figure 6 provides a three dimensional visualization of the scoring for two datasets from prosite, PS00541 and PS01024 (see Table 3), with respectively 144 and 5527 contiguous motifs. Additionally, two other datasets were used to evaluate rigid gap motifs, one from prosite PS00056 (see Table 6) with 41 motifs and the other from DILIMOT "Q.RLQ..Q" (see Table 5) with 5371 motifs.

Target motifs can be spotted by following a combination of three criteria: (i) among the ones that are highly ranked in two or three dimensions; (ii) motifs that significantly deviate from the majority of other motifs; (iii) motifs that appear in the border regions of motif clouds. Figure 6, we can see that even when the support ranking of the target motif is not very high, as in the PS00506 and the DILIMOT datasets, the target motifs can easily be identified. When they appear highly conserved, as in PS00541 and PS01024, their identification is straightforward.

A change in the view point of the 3-dimensional plot may help in further identification of interesting motifs.

## Conclusion

In this paper, we have surveyed and categorized 14 motif significance measures. A general and comprehensive evaluation of the measures has been made. Different measures are designed to assess different properties of the motifs. The appropriate measure or set of measures should be selected according to the problem being tackled, the type of extracted motifs and the characteristics of the data.

From the consistency analysis, it was verified that some measures show conflicting information concerning the interest/significance of motifs, while others have a strongly consistent behavior. In such cases, measures can be replaced by others without lost of information. This is especially true for class-based measures which show a strong consistency among them. Particular examples are Correlation, F-Measure and Discrimination Power. In cases where only one score value can be used, and all items of class information are available (*T*_*N*_, *T*_*P*_, *F*_*P*_, *F*_*N*_), the Correlation measure is recommended. The justification lies on the fact that Correlation provides a more balanced use of all class information items. Correlation, F-Measure and Discrimination Power can be used to measure motif over-representation.

Regarding the ranking analysis, the following main conclusion can be drawn: when target motifs are expected to have very similar characteristics, support and support-based measures are the most appropriate. For the identification of weakly conserved target motifs, Z-Score or LogOdd provide the best results since their main criterion is not directly based on the support but rather on how this measure deviates from the expected value. For strongly conserved motifs, any measure based on support like Sensitivity, Information Gain, LogOdd or Z-Score is good enough to highlight the correct motifs. The poor results that, in general, the Pratt measure obtained can be explained by the fact that it does not include the support as a criterion in its calculation. This measure is more adequate to rank motifs with complex and distinct structures but with similar support values.

Support, Information Gain, LogOdd and Z-Score only evaluate the quality of the motifs exclusively with relation to positive information. When negative information is available, Discrimination Power, F-Measure, Correlation and Surprise measure should be considered. By accounting for the two types of information, their assessment of motif over-representation is more consistent.

In order to obtain a more balanced, robust and unbiased motif evaluation, we recommend the combined use of several significance measures. The large number of reported motifs together with this combination may result in difficulties in spotting the most interesting motifs. This can be overcome by considering three desirable properties: the frequency of the motif, the ranking score among the different measures and the information gain of the motif with relation to the remaining ones. Combined with a three-dimensional visualization, such criteria assist the analyst to detect the most interesting motifs.

Considering three hypothetic motif operations: generalization, specialization and length extension, IG is the most sensitive measure. Motif length extension is the operation with the most significant impact over the Information-theoretic measure and the S measure. The PCA analysis over the Prosite dataset confirms the strong consistency between class based measures and that Z-Score and LogOdd have a very similar behavior.

## Availability

Datasets and scripts (Perl Language) are available as additional files (see Additional File 2).

## Authors' contributions

PGF came up with the core idea, developed the scripts, performed the evaluation and wrote the draft of the manuscript. PJA supervised the study and helped improve the manuscript. Both authors have read and approved the manuscript.

## Supplementary Material

Additional File 1 **Correlation Matrices for four different datasets**. Correlation matrices for the 14 measures. The first three figures correspond to the datasets with sensitivity values for the generated motifs of: (a) less than 33%; (b) between 33% and 66%; (c) greater than 66%. Figure (d) corresponds to the evaluation of the prosite motifs. Due to the symmetric nature of the matrices only the lower triangular part is presented. Click here for file

Additional File 2 Scripts and Datasets. This file contains the sequence datasets (synthetic and real) and the evaluated motifs. This data is separated according to the tables where it is presented and discussed. It also contains script programs (in perl language) developed for this work. Click here for file
