# Supplementary material for: Evaluating deterministic motif significance measures in protein databases
Source: Algorithms Mol Biol. 2007 Dec 24;2:16. doi: 10.1186/1748-7188-2-16 (PMC2254621; doi:10.1186/1748-7188-2-16)
Supplement: Additional File 2 — Scripts and Datasets. This file contains the sequence datasets (synthetic and real) and the evaluated motifs. This data is separated according to the tables where it is presented and discussed. It also contains script programs (in perl language) developed for this work. [file 1748-7188-2-16-S2.zip › Scripts_and_Datasets/Scripts/scripts.doc]

In this article we describe some of the base scripts that have been used in the development of the work described in the paper: “*Evaluating Deterministic Motif Significance Measures in Protein Databases*”. Depending on the test/evaluation that was performed variations of these scripts were also used.

All the scripts have been developed by Pedro Gabriel Ferreira ([pedrogabrieldf@gmail.com](mailto:pedrogabrieldf@gmail.com)) and may contain bugs. You can use at your own risk.

The package BioPerl (www.bioperl.org) is required.

SCRIPTS FOR DATA GENERATION

**Title**: Genetic (DNA;RNA; Proteins) Sequence generator

**Script**: generate_random_seq.pl

**Available**:http://search.cpan.org/src/SENDU/bioperl-1.5.2_102/examples/generate_random_seq.pl

**Description**: We have used a version of the script obtained from the set of examples provided in the BioPerl (www.bioperl.org) distribution and available in the link above.

Our version of this script was developed in order to generate protein sequences according to the characteristics (amino acid frequency, number and length) of the input dataset.

**Title**: Generation of Meta Motifs

**Script**: genMetaMotif.pl

**Parameters**: < output filename >

**Description**: This script is used to generate the meta information of motifs this Information consists of (probability, number-gap, TP,TN, FP, FN) all the parameters will be hard coded in the script; Look for their variables to change their values

**Title**: Plants target motifs in the input dataset with a support of 100%

**Script**: motifPlanter.pl

**Parameters**: <input sequence dataset filename > < length of target motif >

**Description**: Takes as input a file with sequences and a motif length W. It generates a motif of length W and plants it in all the sequences (support 100%). Returns the output as a file with the planted motifs (output filename = "planted_" + < input filename >)

**Title**: Extracts the target motifs from the input dataset to satisfy a given support value.

**Script**: motifBySupp.pl

**Parameters**: <input sequence dataset > < output dataset fileName> <target motif> < specified support>

**Description**: This string takes as input a sequence dataset, a motif and a support value (in percentage). Remove the sufficient number of motifs (which have 100% support) to satisfy the input support. This script is used in complement with the previous script to generate dataset with less frequent contiguous target motifs.

**Title**: Retrieves the support and the frequency (total count) of the motifs in the input dataset

**Script**: motifCount.pl

**Parameters**: <input sequence dataset > <target motif>

**Description**: This script is used to count the number of occurrences of a rigid motif in a file with fasta sequences:

**usage**: perl motifCount.pl file.fa(sta) motif

SCRIPTS FOR MOTIF AND MEASURE EVALUATION

**Title**: Ranking evaluation of Information-theoretic measures in contiguous motifs

**Script**: motifEvaluator.pl

**Parameters**: <sequence dataset> and <target motif>

**Description**: Script to generate the rankings of the target motif for the following measures: support, IG, Pratt, Zscore, LogOdds and Info.Probabilities are generated according to the frequency of the amino acids in the input sequences. Takes as input a file with sequences and a target motif. Scans all the sequences with the size of the motif, using a window sliding methodology. Reports the rank of the target motif for the different measures.

**Example usage**: perl motifEvaluator.pl ps000341.fasta IPCCPV

**Title**: Ranking of the target motif in all the measures (14), considering positive and negative datasets.

**Script**: evalAllmeasures.pl

**Parameters**: <target motif> <file with reported motifs> <positive dataset> <negative dataset >

Positive and negative dataset are sequence datasets in fasta format.

< file with reported motifs > corresponds to the reported motifs obtained from the mining algorithms (Teireisias and Pratt).

The motif file should be in Teiresias format:

144	73	PWC 0 3505 0 3619 0 3733 0 3839 0 3953 0 4067 0 ...

182	72	CR.P 0 76 0 190 0 304 0 418 0 532 0 646 0 760 0 ...

...

See file (motifs_ps00021.txt) for an example.

**Description**: Rank the dataset of motifs with three types of measures, which are:

support, IG, Pratt, Zscore, LogOdds, S, J, I, Sn, Sp, PPV, F, DP, Corr, FPR, Info.

Uses positive and negative datasets.
