# Supplementary material for: Evaluating deterministic motif significance measures in protein databases
Source: Algorithms Mol Biol. 2007 Dec 24;2:16. doi: 10.1186/1748-7188-2-16 (PMC2254621; doi:10.1186/1748-7188-2-16)
Supplement: Additional File 2 — Scripts and Datasets. This file contains the sequence datasets (synthetic and real) and the evaluated motifs. This data is separated according to the tables where it is presented and discussed. It also contains script programs (in perl language) developed for this work. [file 1748-7188-2-16-S2.zip › Scripts_and_Datasets/Scripts/scripts.htm]

In this article we describe some of the base scripts that have been used
in the development of the work described in the paper: “Evaluating
Deterministic Motif Significance Measures in Protein Databases”


In this article
we describe some of the base scripts that have been used in the development of
the work described in the paper: “*Evaluating
Deterministic Motif Significance Measures in Protein Databases*”. Depending
on the test/evaluation that was performed variations of these scripts were also
used.

All the scripts
have been developed by Pedro Gabriel Ferreira (pedrogabrieldf@gmail.com) and may
contain bugs. You can use at your own risk.

The package BioPerl (www.bioperl.org) is required.

SCRIPTS FOR DATA GENERATION

**Title**: Genetic (DNA;RNA; Proteins)
Sequence generator

**Script**: generate\_random\_seq.pl

**Available**:http://search.cpan.org/src/SENDU/bioperl-1.5.2\_102/examples/generate\_random\_seq.pl

**Description**: We have used a version of the script obtained
from the set of examples provided in the BioPerl
(www.bioperl.org) distribution and available in the link above.

Our version of this script was developed in order to
generate protein sequences according to the characteristics (amino acid
frequency, number and length) of the input dataset.

**Title**: Generation of Meta
Motifs

**Script**: genMetaMotif.pl

**Parameters**:  <
output filename >

**Description**: This script is used to generate the meta
information of motifs this Information consists of (probability, number-gap, TP,TN,
FP, FN) all the parameters will be hard coded in the script; Look for their
variables to change their values

**Title**: Plants target motifs in the input dataset with a support
of 100%

**Script**: motifPlanter.pl

**Parameters**: <input sequence dataset filename > <
length of target motif >

**Description**: Takes as input a file with sequences and a
motif length W. It generates a motif of length W and plants it in all the
sequences (support 100%). Returns the output as a file with the planted motifs
(output filename = "planted\_" + < input filename >)

**Title**: Extracts the target motifs from the input dataset to
satisfy a given support value.

**Script**: motifBySupp.pl

**Parameters**: <input sequence dataset > < output
dataset fileName> <target motif> <
specified support>

**Description**: This string takes as input a sequence dataset,
a motif and a support value (in percentage). Remove the sufficient number of
motifs (which have 100% support) to satisfy the input support. This script is
used in complement with the previous script to generate dataset with less
frequent contiguous target motifs.

**Title**: Retrieves the support and the frequency (total count) of
the motifs in the input dataset

**Script**: motifCount.pl

**Parameters**: <input sequence dataset > <target
motif>

**Description**: This script is used to count the number of
occurrences of a rigid motif in a file with fasta
sequences:

**usage**: perl motifCount.pl file.fa(sta) motif

SCRIPTS FOR MOTIF AND MEASURE
EVALUATION

**Title**: Ranking evaluation of Information-theoretic measures in
contiguous motifs

**Script**: motifEvaluator.pl

**Parameters**: <sequence dataset> and <target
motif>

**Description**: Script to generate the rankings of the target
motif for the following measures: support, IG, Pratt, Zscore,
LogOdds and Info.Probabilities
are generated according to the frequency of the amino acids in the input
sequences. Takes as input a file with sequences and a target
motif. Scans all the sequences with the size of the
motif, using a window sliding methodology. Reports the rank of the
target motif for the 
different measures.

**Example usage**: perl
motifEvaluator.pl ps000341.fasta IPCCPV

**Title**: Ranking of the target motif in all the measures (14),
considering positive and negative datasets.

**Script**: evalAllmeasures.pl

**Parameters**: <target motif> <file with reported
motifs> <positive dataset> <negative dataset >

Positive and negative dataset are sequence datasets in fasta format.

< file with reported motifs
> corresponds to the reported motifs obtained from the mining algorithms (Teireisias and Pratt).

The motif file should be in Teiresias
format:

144      73        PWC 0 3505 0 3619 0 3733 0 3839 0 3953 0
4067 0 ...

182      72        CR.P 0 76 0 190 0 304 0 418 0 532 0 646
0 760 0 ...

...

See file (motifs\_ps00021.txt) for an example.

**Description**: Rank the dataset of motifs with three types of
measures, which are:

support, IG, Pratt, Zscore, LogOdds, S, J, I, Sn, Sp, PPV, F,
DP, Corr, FPR, Info.

Uses positive and negative datasets.
